# Folie à Trois à Lubumbashi: à propos d’un cas

**DOI:** 10.11604/pamj.2013.14.47.2339

**Published:** 2013-02-04

**Authors:** Marcellin Bugeme, Olivier Mukuku, Pitchou Mobambo, Bora Koba, Emmanuel Muyumba

**Affiliations:** 1 Hôpital Jason Sendwe, Lubumbashi, RDC; 2 Centre Neuro-Psychiatrique Dr Joseph Guislain, Lubumbashi, RDC; 3 Cliniques Universitaires de Lubumbashi, RDC

**Keywords:** Folie à trois, trouble psychotique partagé, délires induits, délires collectifs, Folie à trois, shared psychotic disorder, induced delusions, collective delusions

## Abstract

Le trouble psychotique partagé est une entité psychiatrique rare caractérisée par la transmission des idées délirantes d’un patient dit “inducteur”, à un autre dit “induit”, tous vivant en association proche, dans un milieu clos et isolé, partageant des idées délirantes sur le même thème. Nous vous rapportons un cas clinique de “folie à trois” mettant en jeu un délire de persécution à mécanisme hallucinatoire et interprétatif par trois soeurs de même famille biologique, orphelines de père, vivant ensemble, ayant un niveau socio-économique bas après la mort de leur père. Elles ont des relations étroitement liéeset font leurs activités ensemble. L’aînée étant parvenue à partager ses idées délirantes avec ses soeurs et toutes troisse sont retrouvées dans un état d’excitation psychomotrice.

## Introduction

Le trouble psychotique partagé est une entité psychiatrique caractérisée par des idées délirantes semblables partagées par deux ou plusieurs personnes qui ont une relation proche. Il a été d′abord décrit par Jules Baillarger en 1860, qui a nommé cette condition comme “folie communiquée”. Il a été différemment appelé: psychose d′association, trouble paranoïde partagé, la folie contagieuse. C’est en 1877 que Lasègueet Falret ont puinventer le terme “folie à deux” ou “psychose de deux” [1, 2].

Sa caractéristique principale est la transmission des idées délirantes d’une personne qui a déjà un trouble psychotique avec des idées délirantes avérées à une autre personne en association étroite. Cette personne peut induire entièrement ou en partie ses idées à une autre personnequi va développer des idées semblables. Le contenu des idées délirantes partagées peut dépendre du diagnostic de l’inducteur [3]. Selon que les idées délirantes sont partagées parmi deux, trois ou quatre personnes, on parle respectivement de folie à deux, folie à trois ou folie à quatre.Rarement tous les membres de la famille partagent les mêmes illusions et ceci est appelé folie à famille [4, 5].

Le cas que nous vous rapportons inclut des idées délirantes partagées parmi trois soeurs et c’est le premier rapporté dans notre milieu.

## Patient et observation

Il s’agit d’un cas clinique de trouble psychotique partagé impliquant trois soeurs de mêmes parents biologiquesvivant ensemble avec leur mère et orphelines de père depuis dix ans. Aucune d’entre elles n’avait jamais présenté une quelconque maladie psychique et depuis leur enfance, elles n’avaient jamais présenté un retard lié au développement ou une défiscience intellectuelle ou d’autres problèmes significatifs au cours de leur enfance ou de leur scolarisation. Elles n’ont jamais été victimes de violence psychologique ou physique (violence sexuelle) et aucune d’entre elles ne présente une affection médicale générale. Elles ne consomment pas des boissons alcoolisées ni toute autre substance psychoactive. Sur le plan financier, elles vivent à un niveau bas depuis la mort de leur père.

soeur A, âgée de 37 ans et ainée de trois, est amenée au centre psychiatrique en agitation psychomotrice avec sesdeux soeurs présentant le même tableau qu’elle. Ceci évolue depuis environ trois semaines après qu’elle ait eu une vision (de part de Dieu) qu’elle a partagée à ses deux jeunes soeurs. Emportées par les mêmes idées qu’elle, ces dernières s’isoleront pour jeûner et prier pendant plusieursjours avec elle et là elles recevront d’autres visions venant de Dieu. Finalement,elles s’agiteront et une foule immense viendra s’attrouper autour d’elles;elles devenaient agressives etémettaient des injures à l’égard de la foule. Après quoi, elles furent amenées par leur famille au centre neuropsychiatrique Dr Joseph Guislain pour être prises en charge.

Toutes les trois ne rapportent qu’unehistoire identique prétendant qu’elles sont toutes poursuivies par certains membres de leur famille qui cherchent à leur donner la mort sans toutefois préciser de qui s’agit-il. Elles ajoutent endisant qu’elles ont reçues des visions de la part de Dieu leur demandant qu’elles se rendent auprès de leur belle-soeur car elle pratiquait la sorcellerie et qu’elle est à l’origine de leur souffrance soulignant que rien nemarche dans la famille sur le plan financier depuis le décès de leur père. Elles finissent endisant que, dans leur vision, elles voient ressuscitée leur soeur décédée depuis plusieurs années et qu’une foule immense s’était accourue vers elle et l’ont tabassée à mort.

soeur A est célibataire et mère de 3 enfants et est diplômée d’université en Relations internationales. Elle est décrite comme étant extravertie. Son observation psychiatrique rapporte une activité gestuelle accruefaite des secousses des mains, d’une attitude de prière et du hochement de la tête, un langage logorrhéique, une humeur exaltée et une psychomotricité très instable. La patiente est inconsciente du caractère morbide de son trouble et décrit des hallucinations à thèmes multiples, à mécanisme intuitif et hallucinatoire audiovisuel et les réactions étaient l’autoagressivité.

Concernant la soeur B, elle est âgée de 31 ans, célibataire, mère de un enfant.Elle est en première année de licence en informatique de gestion.L’examen psychiatrique relève un semi-mutisme, un contact laborieux, une stéréotypie gestuelle faite de bouchage d’oreilles avec ses mains et d’élévation des mains en signe de prière et d’exorcisme, une psychomotricité accrue. Sa tenue corporelle et vestimentaire était négligée et elle avait une humeur triste avec pleurs et larmes coulant constamment. Elle était également inconsciente de la morbidité de son trouble et rapportait des idées délirantes mystico-religieuses mal systématisées avec réactions multiples.

Quant à la soeur C, elle est âgée de 26 ans, diplômée d’Etat (en section Biologie-chimie), célibataire et sans enfant. Elle est décrite comme étant calme.L’observation psychiatrique note un langage logorrhéique, une activité gestuelle accrue, des cheveux sales et non peignés, une tenue négligée, une humeur colérique et également une psychomotricité accrue. Elle est aussi inconsciente du caractère morbide du trouble. Elle présentait des délires de persécution à mécanisme hallucinatoire.

Après entretien avec les trois soeurs, le diagnostic de délire collectif à trois avait été confirmé. Elles ont été hospitalisées séparément chacune dans sa chambre et un traitement psychopharmacologique fait de Halopéridol (15mg par jour), de Lévomépromazine (150mg par jour) et de Chlorpromazine (100mg par jour) leur avait été administré au cours de leur hospitalisation.

Après une semaine d’hospitalisation, il y a eu une nette amélioration de leur état clinique et toute leur symptomatologie avait complétement disparu mais notons que la vitesse de la régression des symptômes était lente chez la soeur A comparativement à ses deux soeurs. Elles ont pu passer une semaine de plus en hospitalisation sans médicaments pour un suivi permanent et une rémission complète avait été noté. Après leur sortie de l’hôpital, elles ont bénéficié d’un contrôle régulier en ambulatoire pendant une période de 6 mois et aucun trouble n’avait été observé.

## Discussion

Le trouble psychotique partagé ou la folie partagée est une entité psychiatrique considérée comme rare. Les premières définitions furent apportées par Lasègue et Falret en 1877: deux sujets, vivant en association proche, dans un milieu clos et isolé, partagent des idées délirantes sur le même thème [1, 2]. Son épidémiologie est assez mal connue et la plupart des données sont basées sur des simples rapports de cas. Peu d’informations systématiques sur la prévalence du trouble psychotique partagé sont disponibles.Trois enquêtes qui ont passé en revue de toutes les présentations de cas sur cette pathologie indiquent que 242 cas ont été publiés de 1877 à 2005 [6–8]. Des données limitées suggèrent que le trouble psychotique partagé est légèrement plus fréquent chez les femmes que chez les hommes [3, 8].

Cette entité clinique a donc la particularité de mettre en jeu, non pas un, mais plusieurs sujets; le tableau regroupe un premier patient authentiquement délirant, en général paranoïaque, et un deuxième sujet (voire un groupe de sujets) qui n′est pas de structure psychotique. Ce deuxième sujet, voire le troisième à la faveur d′une situation de confinement particulier et d′une conjoncture qui exclut bien souvent toute influence extérieure, va ou vont progressivement adopter la conviction délirante de la personne inductrice dont ils partagent la vie [2]. Dans notre cas, c’était la même situation que nous avons pu observer chez la soeur B et la soeur C qui sont les personnes induites, elles ont fait sienne la certitude de la soeur A, reprenant un à un les termes de ses délires et hallucinations qui lui étaient propres.

L’étude de Lasègue et Faret relève trois conditions régissant le trouble psychotique partagé. Premièrement, la loi de la différence intellectuelle et de caractère. Deuxièmement, la loi du milieu clos implique que les individus partagent absolument tout, en dehors de la moindre influence du monde extérieure. Et troisièmement, la loi de la vraisemblance selon laquelle le délire doit être vraisemblable, de manière à ce que la conviction délirante puisse être communiquée entre les différents individus [1–3].

Les individus qui finissent par partager des croyances délirantes sont souvent liés par le sang ou le mariage et vivent ensemble depuis longtemps, parfois dans un isolement social relatif.L′individu source est généralement plus vieux, plus intelligent, mieux instruit et a des traits de personnalité plus forts et les délires qui sont surtout des idées de persécution [3]. Dans le cas présent, l′individu psychotique (soeur A) manifeste des caractéristiques semblables, c’est-à-dire qu’elle est de six ans plus âgée que la deuxième (soeur B) et de 11 ans plus âgée que la troisième (soeur C); elle a un niveau d’instruction plus élevé par rapport à ses deux soeurs. Ces patientes sont de mêmes parents biologiques, elles habitent et font toutes leurs activités ensemble.

La principale mesure thérapeutique préconisée par les aliénistes qui ont initialement décrit ce tableau clinique, consiste à séparer les patients. Celui des patients qui n′est pas psychotique renonce alors assez rapidement à cette croyance d′allure délirante et retrouve son assise symbolique antérieure, tandis que le patient principal maintient intacte sa conviction [1, 2]. Dans le cas que nous rapportons les trois soeurs ont été séparées et hospitalisées dans des chambres différentes et les soeurs considérées comme induites ont été les premières à se stabiliser. Mais il faut noter qu’il n’est pas toujours évident que la seule séparation puisse permettre le rétablissement du sujet induit; c’est pourquoi, comme le suggèrent d’autres auteurs, nous y avonsaussi adjoint un traitement psychopharmacologique [9, 10].

## Conclusion

Dans ce cas rapporté, la patiente principale ou l’inductrice qui a réussi à partager ses idées délirantes à ses deux soeurs avait une position très forte dans sa famille et était plus instruite que les patientes induites ou secondaires qui, elles, ont une personnalité dépendante. Le fait qu’elles soient des mêmes parents biologiquesetqu’elles aient des relations étroitement liées entre elles, a favorisé le développement du trouble psychotique non seulement chez la deuxième, mais aussi chez la troisième soeur. C’est un cas rare et c’est le premier que nous ayons pu observer dans notre milieu.

## References

[1] Lasegue C, Falret J (1877). La folie à deux (ou folie communiquée). Annales Medico-Psychologiques..

[2] Regis E (1880). La folie à deux ou folie simultanée avec observations recueillies à la clinique de Pathologie mentale.

[3] American Psychiatric Association (2005). Manuel Diagnostique et Statistique des Troubles Mentaux (DSM-IV-TR)..

[4] Erol A, Ersoy B, Gulpek D, Mete L (2008). Folie a famille: case report. Anatolian Journal of Psychiatry..

[5] Srivastava A, Borkar HA (2010). Folie a famille. Indian J Psychiatry..

[6] Gralnick A (1942). Folie a deux - the psychosis of association - A review of 103 cases in the entire English literature: withcase presentations. Psychiatric Q..

[7] Silveria JM, Seeman MV (1995). Shared psychotic disorder: a critical review of the literature. Can J Psychiatry..

[8] Arnone D, Patel A, Tan GM (2006). The nosological significance of folie a deux: a review of the literature. Ann Gen Psychiatry..

[9] Suresh Kumar PN, Subramanyam N, Biju T, Abu A, Kishore K (2005). Folie à deux. Indian J Psychiatry..

[10] Jana AK, Praharaj SK, Sarkar S, Dotivala KN, Chabungbam G (2009). Folie á deux between two unrelated individuals. Turk PsikiyatriDerg..

